# Development and psychometric evaluation of a women shift workers’ reproductive health questionnaire: study protocol for a sequential exploratory mixed-method study

**DOI:** 10.1186/s12978-018-0456-0

**Published:** 2018-02-06

**Authors:** Maryam Nikpour, Aram Tirgar, Abbas Ebadi, Fatemeh Ghaffari, Mojgan Firouzbakht, Mahmod Hajiahmadi

**Affiliations:** 1 Social Determinants of Health Research Center, Health Research Institute, Babol University of Medical Sciences, Babol, Iran; 2 Behavioral Sciences Research Center, Nursing Faculty, Baqiyatallah University of Medical Sciences, Tehran, Iran; 3 Nursing Care Research Center, Health Research Institute, Babol University of Medical Sciences, Babol, Iran; 4 Student Research Committee, Babol University of Medical Sciences, Babol, Iran; 5 Non Communicable Pediatric Disease Research Center, Health Research Institute, Babol University of Medical Sciences, Babol, Iran

**Keywords:** Reproductive health, Shift workers, Psychometric evaluation, Sequential exploratory mixed-method study, Validity, Reliability, Women shift workers’ reproductive health questionnaire, WSW-RHQ

## Abstract

**Background:**

Although shift works is a certain treat for female reproductive health, but currently, there is no standardized instrument for measuring reproductive health among female shift workers. This study aims to develop and evaluate the psychometric properties of a Women Shift Workers’ Reproductive Health Questionnaire (WSW-RHQ).

**Methods:**

This is a sequential exploratory mixed-method study with a qualitative and a quantitative phase. In the qualitative phase, semi-structured interviews will be held with female shift workers who live in Mazandaran Province, Iran, additionally, the literature review will be performed by searching electronic databases. Sampling will be done in different workplaces and with maximum variation respecting female shift workers’ age and job and educational and different economic situation. Interview data will be analyzed using conventional content analysis and then, the primary item pool for the questionnaire will be developed. In the quantitative phase, we will evaluate the psychometric properties of the questionnaire, i.e. its face, content, construct as well as reliability via the internal consistency, stability. Finally, a scoring system will be developed for the questionnaire.

**Discussion:**

The development of WSW-RHQ will facilitate the promotion and implementation of reproductive health interventions and assessment of their effectiveness. Other scholars can cross-culturally adapt and use the questionnaire according to their immediate contexts.

## Plain English summary

‘Shift work’ is a work schedule involving irregular or unusual working hours (arbitrarily between 6 pm and 7 am), compared to a normal daytime work schedule. Most female workers in Iran work in the service sector, particularly healthcare organizations, welfare institutes, and nursing homes. In these settings, workers are required to work as per shift work.

Shift work is associated with a wide range of physical, psychological, and social health problems. It can affect different aspects of women’s reproductive health. For example, women may experience problems in their marital and sexual relationships as well as their menstrual cycle. For all women, reproductive health is very important. Since there is no standardised instrument for measuring reproductive health among female shift workers, we plan to design a Female Shift Workers’ Reproductive Health Questionnaire to do so.

This study will be completed in two phases. In the first phase, an initial questionnaire will be conducted by way of interviews among married female shift workers. Respondents would include all female shift workers living in Mazandaran province, Iran, and working in hospitals, factories, student dormitories, and welfare and rehabilitation centres. In the second phase, the initial questionnaire will be conducted among some specialists and 400 women shift workers in order to determine validity and reliability of the questionnaire. We hope that it will be a valid and reliable instrument to assess the effects of shift work on women’s reproductive health.

## Background

Around half of the women in the world are working [1]. Although female employment rate in our country, Iran, is lower that the global average, it has increased from 14% in 2000 to 18% in 2014 [2]. Most female workers in Iran work in the service sector [3], particularly healthcare organizations [4], welfare institutes, and nursing homes. In these settings, workers need to provide services throughout day and night via doing shift work [5].

Shift work disrupts normal circadian rhythm, reduces blood level of melatonin, causes sleep-wake cycle disorders [3], and alters pituitary production of prolactin [6] and sexual hormones such as luteinizing [7] and estrogen [8]. These alterations cause menstrual irregularities and thereby threaten women’s reproductive health [3, 9]. Moreover, due to working in different shifts and even in holidays, female shift workers are departed from their families and husbands and hence, they may experience problems in their marital and sexual relationships [10]. Attarchi and et al. compared daytime female and shift workers and found that the blood level of prolactin and the rate of menstrual irregularities among shift workers were significantly higher than daytime workers [11]. White & Keith also studied 1668 married women and men reported shift work as a negative significant factor behind the quality of marital relationship [12]. Moreover, Marino et al. and Zhu et al. reported that shift work can affect different aspects of women’s reproductive health [13, 14].

There is most famous instrument for measuring the effects of shift work. The Survey of Shift work (SOS) is a valid instrument which assesses the effects of shift work on physical and mental health as well as personal, familial, and social relationships [15, 16]. Moreover, previous studies developed different instruments for reproductive health measurement, among which are the Health Assessment Toolkit for Conflict-Affected Women [17], Sexual and Reproductive Health Needs Assessment among Mobile and Vulnerable Population Questionnaire [18], and Youth Reproductive Health Questionnaire [19]. However, none of these questionnaires are appropriate for reproductive health assessment among female shift workers. Therefore, previous studies either used non-standardized instruments for this purpose (did not evaluate contend and construct validity) [20, 21] or assessed only one aspect of reproductive health such as the process of menstruation [22], sexual satisfaction [23], pregnancy outcomes [24, 25], and infertility [26]. Given the lack of standardized instruments for reproductive health assessment among female shift workers, the present study aims to develop and evaluate the psychometric properties of a women Shift Workers’ Reproductive Health Questionnaire (WSW-RHQ).

This study will be done in a qualitative and a quantitative phase (See the Methods section).

### Objectives

The objectives of each phase are as follows.

*The objectives of the qualitative phase:*

1. Defining the concept of female shift workers’ reproductive health;
2. Exploring the main constructs and sub-constructs of the concept; and
3. Developing a comprehensive item pool for WSW-RHQ.

*The objectives of the quantitative phase:*

1. Assessing the face and the content validity of WSW-RHQ;
2. Assessing the construct validity of WSW-RHQ;
3. Assessing the convergent and the divergent validity of WSW-RHQ;
4. Assessing the reliability of WSW-RHQ using the internal consistency and the stability assessment methods;
5. Assessing the feasibility of WSW-RHQ use;
6. Determining the floor and the ceiling effects; and
7. Developing WSW-RHQ scoring system.

## Methods

**Fig. 1:**
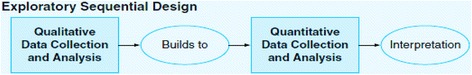
The Schematic diagram of a sequential exploratory mixed-method study

This is a sequential exploratory mixed-method study. Sequential exploratory mixed-method is the design of choice when a researcher does not know the most important concepts for the study and also when there are no appropriate tools for the measurement of an intended concept [27]. Sequential exploratory mixed-method studies have a qualitative and a quantitative phase (Fig. 1).

**Fig. 2:**
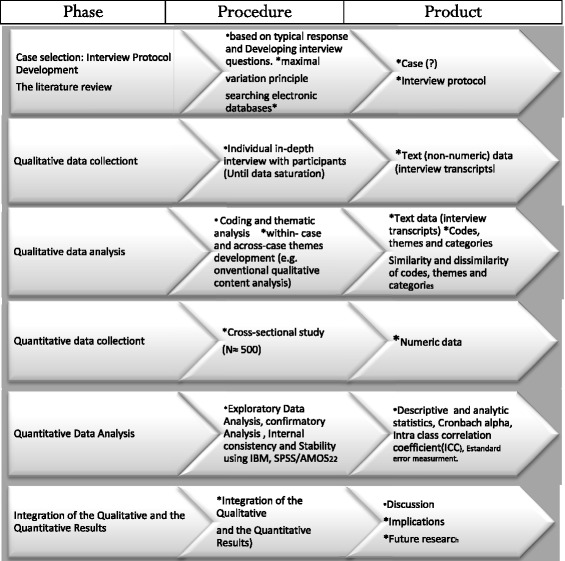
Diagram. Different phases and steps of the study

In the qualitative phase, the concept of reproductive health and its dimensions will be explored based on female shift workers’ experiences and the literature review and then, the primary items of WSW-RHQ are developed. After that, the psychometric properties of the questionnaire will be assessed in the quantitative phase (Fig. 2).

### The qualitative phase

#### Data collection

For data collection, the inductive method (codes extracted from semi-structured personal interviews) and deductive (codes extracted from the literature review) will be used. Semi-structured personal interviews will be done with female shift workers. An interview guide is used for the interviews [28]. The guide will be developed through consulting experts in qualitative research and reproductive health. Primarily, the aims of the study and of the interviews will be explained to participants and their informed consents will be secured. Each interview will be opened by asking an open-ended question about the intended interviewee’s reproductive health status and the effects of shift work on her reproductive health. Next questions will be asked based on her answers to the first question and also based on the interview guide. Whenever needed, we will also use probing questions such as “What do you mean by this?” “Can you provide more detailed explanations?” “What did you feel about this topic?” Moreover, at the end of each interview, the interviewee will be allowed to speak about any missed points. All interviews will be recorded and immediately transcribed word by word. During interviews, participants’ nonverbal messages (such as tone, silence, emphasis, cry, and sigh) will also be documented. Additionally, semi-structured personal interviews, the literature review will be performed to clarify the concept and dimensions of reproductive health.

#### Data analysis

As soon as the first interview is done and transcribed, data analysis will be started using conventional content analysis with the Grameim and Lundman model. The transcript of each interview will be read frequently to determine meaning units, i.e. key sentences and words which relate to the aim of study. The units will be interpreted and coded [29]. Then, the codes will be compared and categorized into subcategories according to their conceptual similarities and differences. Subcategories will also be grouped together based on their interrelationships in order to form comprehensive, mutually exclusive categories. Finally, the main categories and subcategories of female shift workers’ reproductive health are identified. These main categories and subcategories will be used to generate the primary item pool for WSW-RHQ. The existing literature and instruments will also be used for item generation.

#### Sample size and sampling strategy

Study population consists of all female shift workers living in three cities in Mazandaran province, Iran, namely Qaemshahr, Amol, and Babol. These cities are located in the center of Mazandaran province and are representative of the culture and people in the province. Therefore, the data collected from these cities are more generalizable to the people in other cities of the province. Sampling will be done with maximum variation respecting female shift workers’ age and job and educational and different economic situation. Moreover, women will be recruited from different workplaces (such as hospitals, factories, student dormitories, welfare and rehabilitation centers, and nursing homes). In qualitative studies, sample size cannot be predetermined; rather it is determined based on the time of data saturation, i.e. when data collection produces no new finding [30]. Inclusion criteria are consent for participation, an age of 18–49, a shift work experience of 2 years or more, no pregnancy at the time of the study, no history of hospitalization for psychiatric disorders, and no significant losses during the past 6 months before the study.

### The quantitative phase

The focus of this phase is on assessing the psychometric properties of WSW-RHQ, i.e. its face, content, construct, divergent, and convergent validity as well as reliability.

#### Face validity assessment

Qualitative and quantitative methods will be used for face validity assessment. In the qualitative step, female shift workers will be asked to comment on the difficulty, appropriateness, and clarity of the items [31]. Quantitative face validity assessment will be done via the item impact measurement technique. Accordingly, ten female shift workers will score the importance of each item from 1 (“Lowest importance”) to 5 (“Highest importance”). Then, the impact score of each item is calculated through multiplying the mean importance score of that item by then, i.e. the number of scoring women. Item impact scores greater than 1.5 are acceptable [32].

#### Content validity assessment

Content validity will also be assessed via qualitative and quantitative methods [33]. During qualitative content validity assessment, ten experts in instrument development, midwifery, reproductive health, psychology, gynecology, and obstetrics will be asked to assess the appropriate wording, grammar, item allocation, and scaling of the items [34]. Moreover, quantitative face validity will be assessed via the content validity ratio (CVR) and content validity index (CVI)) [35]. For CVR calculation, the same experts will be invited to assess item essentiality. Items which are determined by at least nine out of ten experts to be essential will be kept in the questionnaire [36]. On the other hand, CVI will be calculated through inviting the same experts to rate the relevance of each item on a four-point scale from 1 (“Irrelevant”) to 4 (“Completely relevant”) and then, dividing the number of experts who rate that item 3 or 4 by the total number of the experts. CVI values greater than 0.78 are considered acceptable [37]. Also, S-CVI (Average of the I-CVIs for all items on the scale) will be assessed via mean scores for content validity index. S-CVI values of greater than 0.9 indicating that it is acceptable [38].

#### Construct validity assessment

The construct validity of FSWRHQ will be evaluated via exploratory factor analysis.

#### Sample size and sampling strategy

One method for calculating sample size in exploratory factor analysis is the rule of thumb which states that samples greater than 300 are appropriate for most exploratory factor analyses [39]. Therefore, in this study, sample size is considered to be 400. Accordingly, based on the total numbers of female shift workers in hospitals, welfare and rehabilitation centers, nursing homes, student dormitories, and industrial factories, a proportionate number of shift workers will be recruited from each setting through systematic random sampling. Eligibility criteria are the same as those explained in the qualitative phase. Participants who fail to answer more than 11% of WSW-RHQ items will be excluded [40].

Exploratory factor analysis will be done to extract the latent constructs of WSW-RHQ. Sample appropriateness will be evaluated via the Kaiser-Meyer-Olkin (KMO) and the Bartlett’s test of sphericity. KMO values of 0.7–0.8 and 0.8–0.9 are interpreted as acceptable and large sample sizes, respectively [41]. Then, latent constructs will be extracted through the liklihood and varimax rotation. Factor loading will be set at 0.5 and greater and thus, items with communalities lower than 0.5 will be deleted from factor analysis [42].

In the next phase of construct validity assessment, the most common goodness of fit indices will be evaluated using confirmatory factor analysis. These indices include root mean score error of approximation (RMSEA), comparative fit index (CFI), adjusted goodness of fit index (AGFI), minimum discrepancy function divided by degrees of freedom (CMIN/DF), and normal fit index (NFI) [43, 44].

#### Convergent and divergent validity assessments

The construct reliability and the Fornell-Larcker criterion will be used for convergent and divergent validity assessments, respectively. Divergent validity is established when the Average Variance Extracted (AVE) value of each factor is greater than the correlation of that factor with other factors [45].

#### Reliability assessment

WSW-RHQ reliability will be evaluated via the internal consistency, stability, and construct reliability assessment methods. During internal consistency assessment, Cronbach’s alpha values will be calculated for WSW-RHQ and its subscales. Values greater than 0.7 are considered acceptable [46]. Also, we will evaluate McDonald Omega for the internal consistency. Recently, some researchers recommended using McDonald Omega as a measure of internal consistency [47]. Stability will also be evaluated via the Test-retest technique, in which thirty female shift workers will complete WSW-RHQ twice with a two-week interval and then, intraclass correlation coefficient (ICC), will be calculated for the questionnaire and its subscales using the two-way mixed effects method. ICC values which are greater than 0.75 show acceptable stability [48]. Moreover, in confirmatory factor analysis, construct reliability and standard error measurement will be evaluated using Hair and colleagues’ technique. Construct reliability values of greater than 0.7 indicate acceptable reliability [49].

#### Assessing the feasibility of WSW-RHQ

The simplicity of WSW-RHQ will be evaluated through the average time needed for its completion and the percentage of participants who do not respond each item [50]. The average time needed for WSW-RHQ completion will be calculated via measuring the time spent by the first fifty female shift workers on completing it. Moreover, non-response rate will be determined using the data collected for construct validity assessment.

#### Determining the floor and the ceiling effects

The floor and the ceiling effects occur when more than 15% of participants acquire the lowest and the highest possible scores, respectively. These effects reflect that the intended instrument has poor content validity [51]. In this study, the floor and the ceiling effects will be calculated using the data collected for construct validity assessment.

#### Developing WSW-RHQ scoring system

**Fig. 3:**
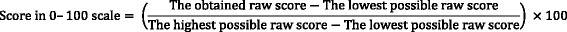
The linear transformation equation

FSWRHQ items will be scored on a five-point Likert-type scale from 5 to 1. Of course, negatively-worded items will be scored reversely, i.e. from 1 to 5. After determining the weight of each item, the standard 0–100 scoring scale will be used. The raw scores of the questionnaire will be converted to 0–100 scores using the linear transformation equation depicted in Fig. 3 [52].

### Statistical data analysis

The SPSS and the AMOS22 will be used for data analysis. Data presentation will be done via the measures of descriptive statistics including mean, standard deviation, and frequencies. Moreover, statistical analyses will be performed through running exploratory factor analysis, Pearson correlation analysis, paired- and independent-sample T-tests, Friedman test, Cronbach’s alpha model, intraclass correlation coefficient, confirmatory factor analysis and standard error measurement.

## Discussion

As a critical step in health-related activities, assessment necessitates the employment of appropriate instruments. WSW-RHQ will create the possibility of collecting data about female shift workers’ reproductive health and measuring the effectiveness of reproductive health interventions.

In this mixed-method study, a questionnaire will be developed based on the definition of reproductive health from the perspectives of female shift workers. Then, the psychometric properties of the questionnaire will be assessed. Given the lack of standardized questionnaires for reproductive health assessment among female shift workers, WSW-RHQ development can be a critical step in assessing and promoting these women’s reproductive health. Other scholars can also cross-culturally adapt and use WSW-RHQ according to their immediate contexts. The strengths of this study are its sequential exploratory mixed-method design, sampling from different cities, and its relatively large sample size. The reluctance of some female shift workers to collaborate with the study may be one of the study limitations.

## Abbreviations

AMOS: Analysis of Moment Structures
EDA: Exploratory Data Analysis
ICC: Intraclass Correlation Coefficient
SPSS: Statistical Package for Social Science
WSW-RHQ: Women Shift Workers’ Reproductive Health Questionnaire
